# Cannabinoid type-1 receptors in CaMKII neurons drive impulsivity in pathological eating behavior

**DOI:** 10.1016/j.molmet.2025.102096

**Published:** 2025-01-07

**Authors:** Elena Martin-Garcia, Laura Domingo-Rodriguez, Beat Lutz, Rafael Maldonado, Inigo Ruiz de Azua

**Affiliations:** 1 Laboratory of Neuropharmacology-Neurophar, Department of Experimental and Health Sciences, Universitat Pompeu Fabra, 08003, Barcelona, Spain; 2 Department of Psychobiology and Methodology in Health Sciences, Universitat Autonoma de Barcelona, 08193, Bellatera, Spain; 3 Hospital del Mar Medical Research Institute (IMIM), Barcelona, Spain; 4 Leibniz Institute for Resilience Research, 55122, Mainz, Germany; 5 Institute of Physiological Chemistry, University Medical Center of the Johannes Gutenberg University, 55128, Mainz, Germany

**Keywords:** Endocannabinoid system, Cannabinoid type 1 receptor (CB1), Impulsivity, Feeding behavior, Obesity, Metabolic syndrome, Food addiction

## Abstract

**Objectives:**

Overconsumption of palatable food and energy accumulation are evolutionary mechanisms of survival when food is scarce. These innate mechanisms becom detrimental in obesogenic environment promoting obesity and related comorbidities, including mood disorders. This study aims at elucidating the role of the endocannabinoid system in energy accumulation and hedonic feeding.

**Methods:**

We applied a genetic strategy to reconstitute cannabinoid type-1 receptor (CB1) expression at functional levels specifically in CaMKII+ neurons (CaMKII-CB1-RS) and adipocytes (Ati-CB1-RS), respectively, in a CB1 deficient background.

**Results:**

Rescued CB1 expression in CaMKII+ neurons, but not in adipocytes, promotes feeding behavior, leading to fasting-induced hyperphagia, increased motivation, and impulsivity to palatable food seeking. In a diet-induced obesity model, CB1 re-expression in CaMKII+ neurons, but not in adipocytes, compared to complete CB1 deficiency, was sufficient to largely restore weight gain, food intake without any effect on glucose intolerance associated with high-fat diet consumption. In a model of glucocorticoid-mediated metabolic syndrome, CaMKII-CB1-RS mice showed all metabolic alterations linked to the human metabolic syndrome except of glucose intolerance. In a binge-eating model mimicking human pathological feeding, CaMKII-CB1-RS mice showed increased seeking and compulsive behavior to palatable food, suggesting crucial roles in foraging and an enhanced susceptibility to addictive-like eating behaviors. Importantly, other contingent behaviors, including increased cognitive flexibility and reduced anxiety-like behaviors, but not depressive-like behaviors, were also observed.

**Conclusions:**

CB1 in CaMKII+ neurons is instrumental in feeding behavior and energy storage under physiological conditions. The exposure to risk factors (hypercaloric diet, glucocorticoid dysregulation) leads to obesity, metabolic syndrome, binge-eating and food addiction.

## Highlights

• CB1 expression exclusively in CaMKII+ neurons, but not in adipocytes, promotes feeding and favors energy accumulation.
• Under risk factors, CB1 function in these neurons increase the vulnerability to diet-, stress-induced obesity and food addiction.
• The restricted expression of CB1 in these neurons was linked to cognitive flexibility, reduced anxiety and despair behavior.
• These findings suggest behavioral traits and mechanisms underlying the onset/origin of pathological feeding behaviors.

## 1 Introduction

The evolution of the human species was determined, among other factors, by an environment of scarcity, where caloric restriction and high physical activity were common living conditions. Thus, neurobiological circuits that govern energy metabolism and feeding behavior to enhance energy storage were genetically advantageous for survival. According to Maslow’s theory, those neurobiological mechanisms that lead to energy storage compete for expression with rival inhibitory behaviors, such as anxiety and fear responses [1], working in coordination with neural circuits that control cognitive functions [2] for a successful survival. Ultimately, multiple needs act in concert to drive an appropriate behavioral outcome and both physiological and contingent motivational needs must be considered concomittantly.

In an obesogenic environment, the evolutionary mechanisms leading to food overconsumption become detrimental, promoting eating-related disorders such as obesity, binge eating disorder (BED) and food addiction. Food overconsumption is a common risk factor in these eating disorders, pointing out a potential underlying mechanism in the development of these pathologies. Obesity has been related to many comorbidities, including mood disorders [3,4], cognitive impairments and increased risk for dementias [2]. Moreover, highly palatable and energy-rich food consumption promotes addictive-like behaviors and BED [5]. Thus, overweight and obese people struggle to control food intake despite the negative health consequences, a behavior analogous to substance use disorders. Even though food addiction differs from obesity, 88% of people meeting the criteria of food addiction following Yale food addiction scale (YFAS 2.0) are obese [6]. Nevertheless, obesity is a multifactorial disorder and food addiction criteria were only achieved by 37% of the German obese population [7]. Additionally, the overlap between food addiction and BED is quite striking, and the classification of YFAS was met by 57% of BED patients [8]. YFAS score was also predictive of emotional dysregulation and depression [8]. Therefore, this evidence highlights a previously underestimated overlap between neuronal circuits that drive survival motivations, including energy balance, anxiety and cognition.

The endocannabinoid (eCB) system is a key regulator of the energy balance through the activation of peripheral and central cannabinoid type-1 receptors (CB1) [[9], [10], [11]]. Overall, the activation of CB1 promotes energy intake and storage through multiple mechanisms [10]. Conversely, constitutive CB1 deletion promotes a lean phenotype in mice under regular chow and high-fat diet (HFD) conditions [12,13]. Importantly, obesity is associated with an elevated eCB tone, which has been involved in the development and maintenance of obesity [14,15]. The eCB system also controls cognitive functions [16] and emotional behaviors [17]. Therefore, the eCB system is a homeostatic mechanism promoting energy storage and resilience to aversive conditions, and allostatic alterations in the eCB system activity can lead to mood and metabolic disorders [[17], [18], [19]]. In fact, pharmacological CB1 blockade in humans by rimonabant was associated with high incidence of major emotional and psychiatric adverse events despite its beneficial metabolic effect in reducing body weight [[20], [21], [22]]. Therefore, it is crucial to decipher anatomically, in which cell types CB1 expression selectively drives a pivotal role in energy balance without affecting rival inhibitory behaviors, such as anxiety and fear responses.

The above evidence suggests that neuronal circuits regulating energy balance are intertwined with those controlling emotion and reward. Hence, a detailed understanding of the neuronal circuits that control these mechanisms is crucial for identifying specific targets to develop novel selective pharmacological treatments allowing to minimize the adverse events. These studies are required to translate preclinical research in animal models to effective therapeutic interventions to tackle eating-related mood disorders. In this study, we analyze the role of the CB1 in CaMKII + neurons and adipocytes, respectively, in the development of obesity, cognitive and emotion related-disorders, BED and food addiction using a genetic rescue approach.

## 2 Materials and methods

### 2.1 Generation of CaMKII-CB1-RS and Ati-CB1-RS mice

To express the CB1 receptor exclusively in CaMKIIα+ or Adiponectin + cells, a rescue approach was applied whereby CB1 receptor function is suppressed globally in Stop-CB1 mice by a IoxP-flanked stop cassette upstream of the endogenous CB1 receptor exon [23]. Crossing Stop-CB1 mice with Cre recombinase-expressing transgenic mice reactivated (i.e., rescued) CB1 receptor function only in Cre-expressing cells. CaMKII-CB1–RS and Ati-CB1-RS mice were generated by mating Stop-CB1 mice with CaMKIIα iCre [24] or AdipoqCreERT2 [25] mice, respectively. 4–5 week-old male mice received tamoxifen (1 mg/mouse, Sigma–Aldrich) once a day, i.p., for five consecutive days. WT controls (WT mice) were generated by crossing Stop-CB1 mice with the general Cre-deleter mouse line EIIa-Cre with CB1 rescue in all cells [23]. Stop-CB1 (CB1–KO) mice were CB1-null mice.

### 2.2 Generation of reporter mouse lines: CaMKII-nuGFP and Ati-nuGFP mice

To generate the reported mouse with labeled fluorescent green nuclei (SUN1-sfGFP-Myc) [26] exclusively in CaMKIIα+ (CaMKII-nuGFP mice) or Adiponectin + cells (Ati-nuGFP mice), we crossed R26-CAG-LSL-Sun1-sfGFP-myc mice with CaMKIIα iCre and AdipoqCreERT2 mice respectively. Ati-nuGFP transgenic and control mice were injected with tamoxifen, as described above.

### 2.3 Mice

Experiments were performed in male mice. Mice (2–5 months old) were housed under conditions of controlled temperature (23 ± 1 °C) and illumination (12-h light/dark cycle). Mice were fed with an SD (13.9 kJ/g, Altromin, C1090/10) or HFD (21.1 kJ/g, Altromin, C1090/60). During HFD or CORT treatment in drinking water, mice were single-housed.

In the binge-eating model, male mice were housed and maintained individually in controlled laboratory conditions (21 ± 1 °C and 55 ± 10% humidity), with food and water available ad libitum during the entire experiment. Mice were tested during the dark phase of a reverse light cycle (lights off at 8.00 a.m. and on at 8.00 p.m.).

All experimental protocols were performed following the guidelines of the European Communities Council Directive 20l0/63/EU and approved by the local ethical committee (Ethical Committee on Animal Care and Use of Rhineland-Palatinate, Germany; Comitè Ètic d’Experimentació Animal-Parc de Recerca Biomèdica de Barcelona, CEEA-PRBB (Protocol Number: RML-16-0048-P1), and Generalitat de Catalunya (Protocol Number: DAAM-9687).

### 2.4 Drugs and treatments

Mice were injected daily i.p. with vehicle [DMSO, Sigma: Tween 80:0.9% saline, 2:1:97 (vol/vol)] or AM6545 (a gift from A. Makriyannis) at 10 mg/kg for two weeks. The CORT treatment in drinking water was conducted as previously described [27,28].

### 2.5 Glucose tolerance test

Glucose and tolerance tests have been described previously [13]. Briefly, overnight-fasted mice were given i.p. glucose (2 mg/g). Tail blood glucose was determined at defined time intervals (OneTouch Ultra, LideScan).

### 2.6 Binge-eating

Mice with similar body weight were randomly divided into two groups (i) basal conditions, ad libitum access to standard diet (SD, 3.52 kcal/g, 75% energy from carbohydrates with 8.3% of sugar, 18% from protein and 7% from fat) for the 7 days of the week (ii) binge-eating conditions with intermittent access to chocolate-mixture hypercaloric food (food was composed of equitable mixed of 4 popular brand chocolate bars highly consumed by humans (MILKA®, SNICKERS®, BOUNTY® AND MARS®) prepared as homogenous food pellets containing 4.92 kcal/g: 52% energy from carbohydrates with 44.4% of sugar, 17% from protein and 24% from fat) and SD for 2 days per week [29,30]. For each 48 h binge-eating cycle, both types of foods were measured at the first 2.5 h (binge behavior), 24 h and 48 h [31].

### 2.7 Experimental sequence

The experimental sequence for the behavioral tests. Initially, mice underwent operant training with chocolate-flavored pellets during 1-h daily sessions to assess the primary reinforcing effects of the pellets over four weeks (from the 5th to the 8th week), as detailed in the self-administration section. Following this, a series of tests were conducted: the light–dark box test (11th week), elevated plus maze (11th week), tail suspension test (12th week), forced swimming test (12th week), and sucrose preference test (18th week), aimed at evaluating anxiolytic- and depressive-like behaviors during intermittent chocolate-mixture diet withdrawal or craving conditions. Additionally, locomotor activity was assessed in the 13th week. Body weight was measured weekly.

Behavioral quantification was conducted through direct observational analysis of video recordings. The duration of each behavioral event was manually recorded by a trained observer using a standard stopwatch, ensuring accurate documentation of the temporal dynamics of the observed behaviors, without the implementation of automated tracking or behavioral analysis software. This traditional observational method allowed for precise temporal measurements while maintaining direct investigator oversight of all behavioral parameters. The specific details of each behavioral test are provided below.

### 2.8 Self-administration

Experiments were performed during the light phase of the dark/light cycle.

#### 2.8.1 Self-administration session

The beginning of self-administration session was signaled by turning on the light for 3 s. Daily sessions maintained by chocolate-flavored pellets lasted 1 h. The self-administration sessions were composed by two pellet periods (25 min) separated by a pellet-free period (10 min). Pellets were delivered contingently after an active response paired with a light stimulus (cue light). A time-out period of 10 s was established after each pellet delivery, where the cue light was off and no reinforcer was provided. During the pellet-free period, no pellet was delivered, and this period was signaled by the illumination of the entire self-administration chamber. In the operant conditioning sessions, mice were under a fixed ratio 1 (FR1, one lever-press resulted in one pellet delivery) schedule of reinforcement followed by an increased FR to 5 (FR5, five lever-presses resulted in one pellet delivery).

*Three addiction-like criteria* were used to evaluate the food addiction-like criteria [32].

#### 2.8.2 Persistence to response

Non-reinforced active responses during the pellet-free period (10 min) on the 3 consecutive days before the progressive ratio (PR).

#### 2.8.3 Motivation

The PR schedule was used to evaluate the motivation. The response required to earn one single pellet escalated according to the following series: 1, 5, 12, 21, 33, 51, 75, 90, 120, 155, 180, 225, 260, 300, 350, 410. The breaking point is the maximum number of lever presses for a single reinforcement. The duration of the PR session was 5 h or no response within 1 h.

#### 2.8.4 Compulsivity

Total number of shocks in the shock test (50 min) were used to evaluate compulsive-like behavior [33,34]. Mice were placed in a chamber without the metal sheet (contextual cue). In this shock-session, mice were under a FR5 schedule with two changes: at the fourth active lever-response received an electric footshock (0.18 mA, 2 s) and at the fifth active lever-response received another footshock with a chocolate-flavored pellet paired with the cue light. The schedule was reinitiated after the time-outperiod or if mice did not perform the fifth response within 1 min.

### 2.9 Behavioral tests

#### 2.9.1 Novelty-suppressed feeding test

Three hours after the onset of the light, 25 pellets of a novel chocolate-flavored food were placed onto the home cage. The latencies to contact and start eating were analyzed. One contact was defined as the snout of the mouse touching the pellets, and eating was identified as licking or biting a pellet. Consumption of the food was measured controlling for spillage, as previously described with some modifications [35].

#### 2.9.2 Light-dark box test

The box consisted of two compartments connected by a tunnel. One compartment was black at 10 lux while the other compartment was white brightly illuminated (500 lux). At the start of the session, mice were placed in the black compartment, head facing a corner. The latency of the first entry into the white compartment, the time spent and the number of entries into both compartments were recorded, as previously described [36].

#### 2.9.3 Elevated plus maze test

The maze consists of a plastic black cross with arms 40 cm long and 6 cm wide placed 50 cm above the floor. Two opposite arms were surrounded by walls (closed arms, 10 lux), while two other arms were devoid of such walls (open arms, 200 lux). Anxiety-like behavior was evaluated as previously described in 5 min [36].

#### 2.9.4 Tail suspension test

Mice were suspended 50 cm above a solid surface using adhesive tape (3/4 of the distance from the base of mouse tail). During a 6 min interval, the total time of immobility was recorded as previously described [36].

#### 2.9.5 Forced swimming test

In a plastic cylinder containing water (27–28 °C), deep enough to prevent touching the bottom of the cylinder and forcing the mouse to swim. The trial lasted 6 min and the total time of immobility after 2 min was recorded. Time of immobility was defined as the animal stopped swimming and only used minimal movements to keep the head above the water, as previously described [36].

#### 2.9.6 Sucrose preference test

Two bottles of water, one with 2% sucrose and the other without, were placed in the cage. Every day for 4 days, the position of the bottles was exchanged and the liquid consumption was measured. On the day 5, the consumption of each bottle (24 h) was recorded. As previously described, the preference for sucrose was calculated as the relative amount of water with sucrose versus total liquid consumed on day 5 [36].

#### 2.9.7 Locomotor activity

Animals were assessed in locomotor activity boxes (Imetronic, Pessac) in low-luminosity (20–25 lux). The locomotor activity was recorded for 60 min as horizontal and vertical activity.

### 2.10 Statistical analysis

All statistical comparisons were performed with SPSS (lBM, version 25). Comparisons between groups were analyzed using Student t-test or U Mann–Whitney depending on the distribution defined by the Kolmogorov–Smirnov normality test. A *P* value < 0.05 was used to determine statistical significance. Outliers (±2 s d. from the mean) were excluded. Several mice suffered dermatitis and were excluded from the experiments.

## 3 Results

### 3.1 Rescue of CB1 in CaMKII + neurons or in adipocytes

We used a genetic rescue approach [23] to selectively re-express CB1 at functional levels in a cell-type specific manner, either in CaMKII + neurons (CaMKII-CB1-RS mice) or in adipocytes (Ati-CB1-RS mice) (Supplementary Fig. 1A). As control groups, we used a global rescue of CB1 (Ella-CB1 as WT mice), and a complete CB1 deletion (Stop-CB1 as CB1–KO mice) (Supplementary Fig. 1A). We validated the selective expression of Cre recombinase in these two cell types, CaMKII + neurons and adipocytes, respectively, by generating two reporter mouse lines, CaMKII-nuGFP and Ati-nuGFP mice (Supplementary Fig. 1B). In Ati-nuGFP mice, nuclear GFP signal was detected in adipocytes (adiponectin + cells), including epididymal white adipose tissue (eWAT) and brown adipose tissue (BAT), but not in any brain region (Supplementary Fig. 1C). We performed qPCR studies in FACS-sorted cells in Ati-nuGFP mice. GFP + cells expressed 4-fold more adiponectin mRNA levels, whereas GFP- cells also expressed 4-fold more Mrc4 mRNA levels, a selective marker of macrophages. In contrast, CaMKII-nuGFP showed nuclei GFP signal exclusively in brain areas, but not in eWAT or BAT (Supplementary Fig. 1C). Additionally, we investigated co-localization of nuclear GFP and GAD65 (a selective marker for GABAergic interneurons) in CaMKII-nuGFP mice. Most of nuGFP signal was detected in non-GAD65+ cells, suggesting that Cre recombinase was mostly expressed in excitatory glutamatergic neurons (Supplementary Figs. 1C–D).

### 3.2 Rescue of CB1 in CaMKII + neurons drives feeding behavior in mice

**Figure 1:**
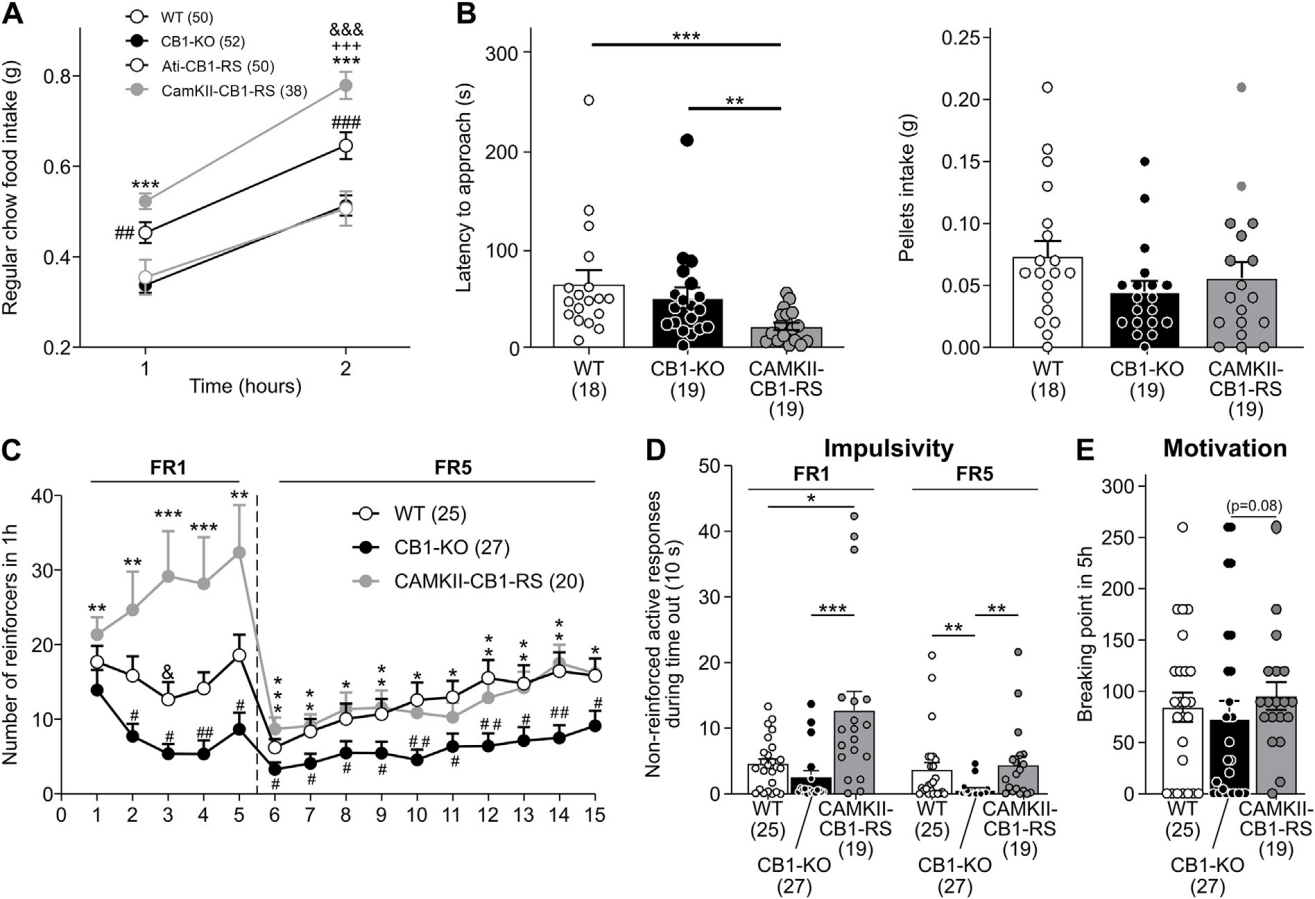
**Feeding behavior of CaMKII-CB1-RS and Ati-CB1-RS mice. (A)** Fasting-refeeding paradigm. Cumulative food intake of regular chow of CaMKII-CB1-RS (gray circles), Ati-CB1-RS (open gray circles), WT (black circles) and CB1–KO (black open circles) mice. Mean ± SEM. 2-way ANOVA, Turkey’s multiple comparisons, ∗∗∗*P* < 0.001 CaMKII-CB1-RS vs CB1–KO; +++*P* < 0.001 CaMKII-CB1-RS vs Ati-CB1-RS; &&&*P* < 0.001 CaMKII-CB1-RS vs WT; ##*P* < 0.01; ###*P* < 0.001 WT vs CB1–KO. **(B)** Novelty-induced feeding inhibition test in WT (white), CB1–KO (black) and CaMKII-CB1-RS (gray) mice. On the left side, latency to approach to novel palatable food. On the right side, palatable food consumption. Mean ± SEM. U Mann–Whitney, ∗∗*P* < 0.01; ∗∗∗*P* < 0.001. **(C)** Operant training to obtain palatable food of CaMKII-CB1-RS (gray circles), WT (black circles) and CB1–KO (white circles) mice. Number of reinforcers in 1 h during the different operant training sessions in a FR1 and FR5 schedule. Mean ± SEM. U Mann–Whitney, ∗*P* < 0.05; ∗∗*P* < 0.01; ∗∗∗*P* < 0.001 CaMKII-CB1-RS vs CB1–KO; &*P* < 0.05 CaMKII-CB1-RS vs WT; #*P* < 0.05; ##*P* < 0.01 WT vs CB1–KO. **(D)** Non-reinforced active responses during the time out period (10 s) after pellet delivery. Mean ± SEM. U Mann–Whitney, ∗*P* < 0.05; ∗∗*P* < 0.01; ∗∗∗*P* < 0.001. **(E)** Breaking point in a 5 h PR schedule. Mean ± SEM. U Mann–Whitney, ∗*P* < 0.05.

In a fasting-refeeding paradigm, the expression of CB1 in CaMKII + neurons increased food intake compared to WT mice in food restriction conditions (Figure 1A). Interestingly, this hyperphagia mainly occurred during the second hour. In contrast, the expression of CB1 in adipocytes resembled the phenotype observed in the total CB1–KO mice, which was significantly lower than in CaMKII-CB1-RS and WT mice (Figure 1A). Therefore, we decided to focus exclusively on CaMKII-CB1-RS mice in the following feeding behavioral tests.

The balance between novelty seeking and risk assessment is instrumental in survival. When mice were exposed to a novelty-suppressed feeding test, CaMKII-CB1-RS mice showed a shorter latency to approach a novel palatable food (Figure 1B) suggesting enhanced drive to incentive properties of palatable food that overcomes anxiogenic behavior to an aversive novel stimulus. No differences were observed in the total consumed food (Figure 1B), suggesting that changes in the metabolic state did not trigger differences.

We also evaluated these mice in an operant paradigm of food addiction [32] (Figure 1C). In a fixed ratio-1 (FR1), CaMKII-CB1-RS mice obtained a higher number of reinforcers compared to the other two genotypes (Figure 1C). An impulsive phenotype was also observed in the rescue mice when the non-reinforced active responses were evaluated during the time-out period after pellet delivery (Figure 1D). However, both WT and rescued mice displayed a similar phenotype, significantly higher than CB1–KO mice, when the responding effort was increased in FR5 (Figure 1C). Accordingly, CaMKII-CB1-RS mice showed a slight non-significant enhanced motivation to obtain rewarding food in comparison to WT and CB1–KO mice in a progressive ratio (PR) schedule (Figure 1E). These results suggest that rescue mice are more prone to obtain rewarding food, although palatable pellets seem equally reinforcing for rescue and WT mice.

### 3.3 CaMKII-CB1-RS mice develop obesity but not glucose intolerance in obesogenic conditions

**Figure 2:**
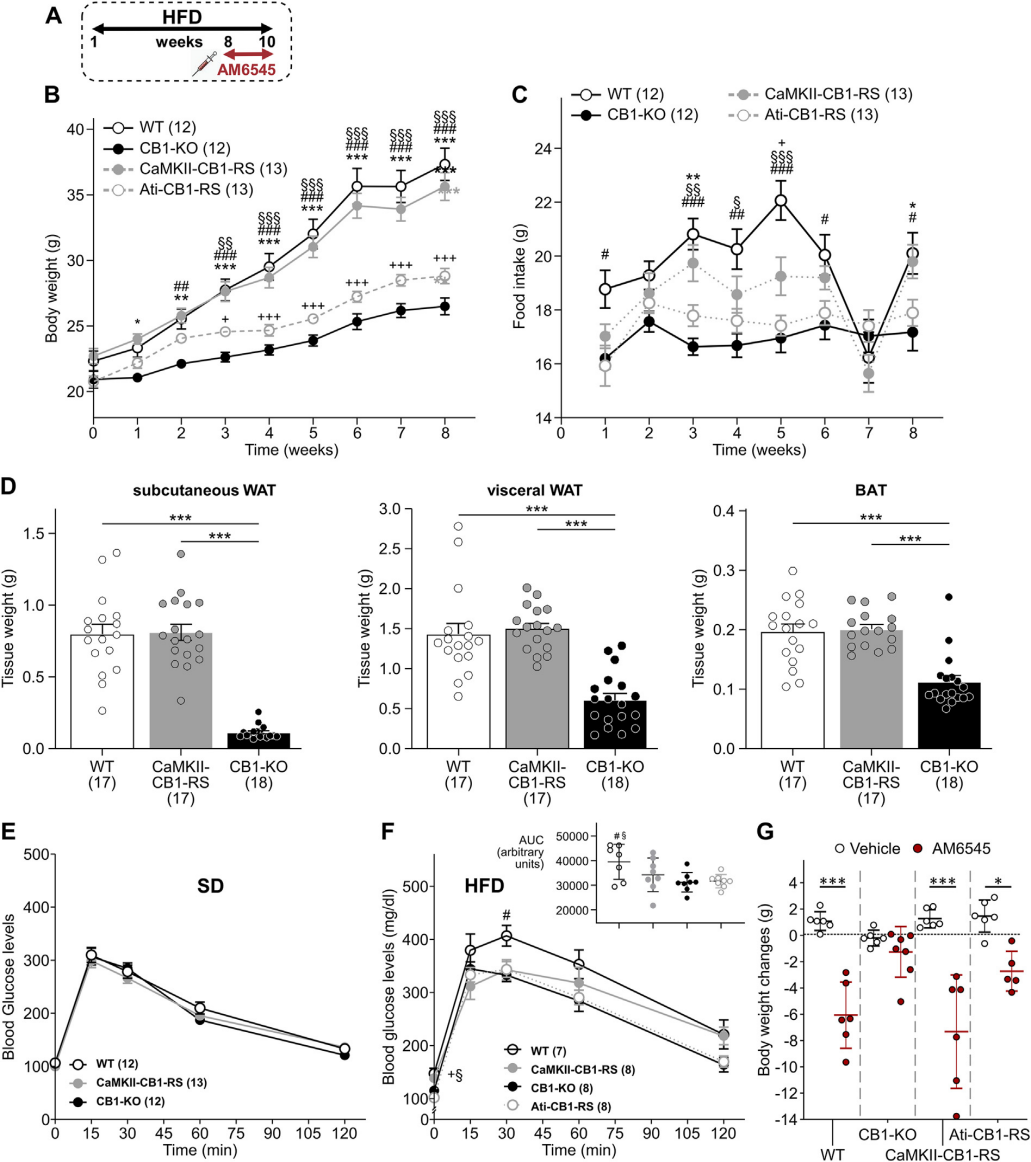
**CaMKII-CB1-RS and Ati-CB1-RS mice under DIO. (A)** Scheme of the experimental design. **(B)** Body weight growth curves and **(C)** food consumption of CaMKII-CB1-RS (gray circles), Ati-CB1-RS (gray open circles), WT (black circles) and CB1–KO (black open circles) mice. Mean ± SEM. 2-way ANOVA, Turkey’s multiple comparisons. ∗*P* < 0.05; ∗∗*P* < 0.01; ∗∗∗*P* < 0.001 CaMKII-CB1-RS vs CB1–KO. +*P* < 0.05; ++*P* < 0.01; +++*P* < 0.001 CaMKII-CB1-RS vs Ati-CB1-RS. ##*P* < 0.01; ###*P* < 0.001; WT vs CB1–KO. §§*P* < 0.01 §§§*P* < 0.001 WT vs Ati-CB1-RS. **(D)** Tissue weight of subcutaneous fat, visceral fat and BAT of CaMKII-CB1-RS (gray), WT (black) and CB1–KO (white) mice. Mean ± SEM. One-way ANOVA, multiple comparison. ∗∗∗*P* < 0.001. **(E)** Glucose tolerance test in SD-fed mice. Mean ± SEM. Two-way ANOVA, Turkey’s multiple comparisons. #*P* < 0.05 WT vs CB1–KO. §*P* < 0.05 WT vs Ati-CB1-RS. +*P* < 0.05 CaMKII-CB1-RS vs Ati-CB1-RS. **(F)** Glucose tolerance test and area under the curve (AUC) of HFD-fed CaMKII-CB1-RS (gray circles), Ati-CB1-RS (gray open circles), WT (black circles) and CB1–KO (black open circles) mice. Mean ± SEM. Two-way ANOVA analysis and Turkey’s multiple comparisons at indicated time points, +*P* < 0.05; CaMKII-CB1-RS vs Ati-CB1-RS. #*P* < 0.05 WT vs CB1–KO. §*P* < 0.05; WT vs Ati-CB1-RS. AUC was analyzed with a one-way ANOVA, #*P* < 0.05; WT vs CB1–KO. §*P* < 0.05 WT vs Ati-CB1-RS. **(G)** Body weight changes after chronic treatment with AM6545 (10 mg/kg, via i.p.) for two consecutive weeks in HFD-fed mice. Mean ± SEM. Unpaired t-test, ∗*P* < 0.05; ∗∗∗*P* < 0.001 AM6545 vs vehicle.

Next, we analyzed CaMKII-CB1-RS and Ati-CB1-RS mice in a diet-induced obesity (DIO) model (Figure 2A). All mice increased body weight over time, but this increase was significantly higher in both CaMKII-CB1-RS and WT mice than in Ati-CB1-RS and full CB1–KO mice (Figure 2B). Nevertheless, Ati-CB1-RS mice still showed a significantly enhanced body weight compared to CB1–KO mice (Figure 2B). Body weight was associated with food consumption in all the genotypes (Figure 2C), except for CaMKII-CB1-RS mice that consumed less amount of hypercaloric HFD than WT mice despite both having similar body weight and adiposity (Figure 2B–D), suggesting additional changes in energy dissipation.

We also performed a glucose tolerance test (GTT) in these mice. No changes in blood glucose levels were found in SD-fed mice in any genotype (Figure 2E), but differences were linked to DIO (Figure 2F). WT mice showed significantly higher glucose levels than Ati-CB1-RS and CB1–KO mice during the test, indicating a glucose intolerance associated with the obese phenotype (Figure 2F). However, despite their elevated body weight, CaMKII-CB1-RS mice were partially protected against glucose intolerance (Figure 2F). Strikingly, the GTT (at the 7th week of HFD) caused a transitory decrease in food intake affecting WT and CamKII-CB1-RS mice but not Ati-CB1-RS and CB1–KO mice (Figure 2C).

The peripheral eCB system was proposed as a potential target in obesity and related disorders [[37], [38], [39]]. Therefore, we treated mice daily with a limited brain prenetrance CB1 antagonist, AM6545 (10 mg/kg, ip), during two weeks, e.g., weeks 8–10. AM6545 treatment significantly decreased body weight and food intake in WT and in the two rescued mouse lines, but not in CB1–KO mice (Figure 2G, Supplementary Fig. 2). We could not observe any effect in weekly food intake in chronic AM6545-treated CB1–KO mice, although an acute effect was previously described [37].

Ati-CB1-RS mice showed a very mild phenotype in feeding behavior and DIO model, hence we only focused on CaMKII-CB1-RS mice in the following experiments.

### 3.4 CaMKII-CB1-RS mice develop metabolic syndrome in a model of stress-related obesity

**Figure 3:**
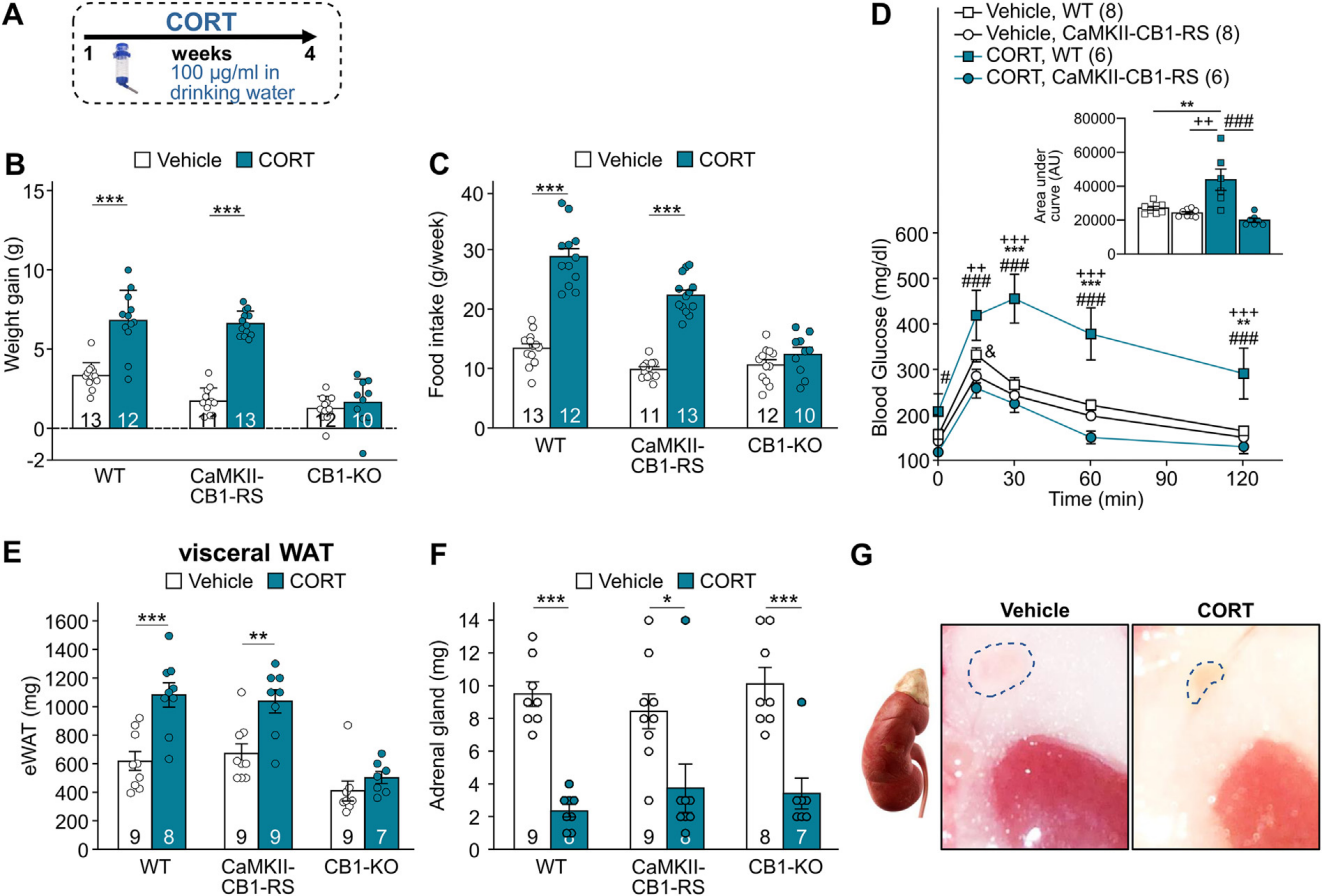
**CaMKII-CB1-RS mice developed metabolic syndrome under chronic CORT treatment. (A)** Scheme of experimental design. **(B)** Weight gain. Mean ± SEM. Unpaired t-test, ∗∗∗*P* < 0.001 CORT vs Vehicle. **(C)** Food intake. Mean ± SEM. Unpaired t-test, ∗∗∗*P* < 0.001 CORT vs Vehicle. **(D)** Glucose levels during a GTT. Mean ± SEM. Two-way ANOVA, multiple comparisons, ∗∗*P* < 0.01; ∗∗∗*P* < 0.001 WT/CORT vs WT/Vehicle. #*P* < 0.05; ###*P* < 0.001 WT/CORT vs CaMKII-CB1-RS/CORT. ++ *P* < 0.01; +++*P* < 0.001 WT/CORT vs CaMKII-CB1-RS/Vehicle. & *P* < 0.05 WT/Vehicle vs CaMKII-CB1-RS/CORT. **(E)** Increased adiposity of visceral fat, **(F)** adrenal size of WT, CaMKII-CB1-RS, and CB1–KO mice. Mean ± SEM. Unpaired *t*-test, ∗*P* < 0.05; ∗∗*P* < 0.01; ∗∗∗*P* < 0.001 CORT vs Vehicle. **(G)** Representative image of adrenal gland of mice treated chronically with vehicle or CORT.

Stress is a risk factor in the development of obesity and metabolic syndrome, and chronically elevated plasma glucocorticoid levels have been strongly associated with fat mass expansion [27,28]. In a model of glucocorticoid-mediated metabolic syndrome, we evaluated the effect of long-term exposure to corticosterone for four weeks (Figure 3A) in CaMKII-CB1-RS, WT and CB1–KO mice. After chronic corticosterone treatment, CaMKII-CB1-RS and WT mice showed largely those alterations related to human metabolic syndrome, including weight gain, visceral fat accumulation and increased food intake, except to glucose intolerance that was exclusively observed in WT mice (Figure 3A–E, Supplementary Fig. 3). These alterations were absent in full CB1–KO mice (Figure 3A–E), highlighting the importance of the eCB system in the development of corticosterone-induced metabolic syndrome. The decreased size and weight of the adrenal gland in corticosterone-treated mice validated the model of glucocorticoid-mediated metabolic syndrome (Figure 3F–G).

### 3.5 CB1 expression in CaMKII neurons was sufficient to promote binge-eating behavior and food addictive-like behavior

**Figure 4:**
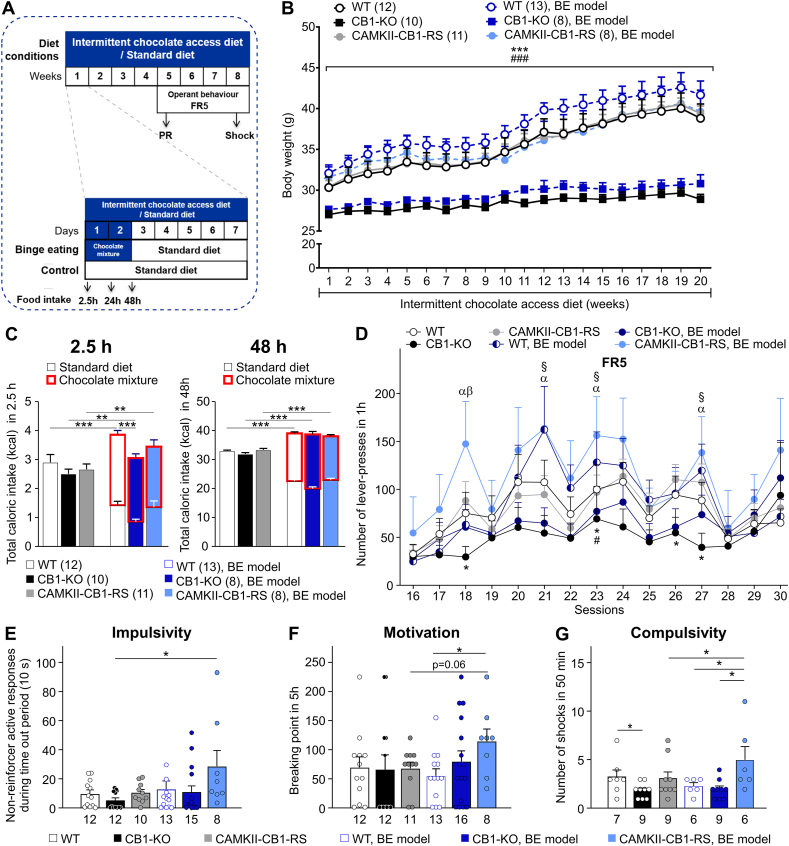
**CB1 expression in CaMKII neurons was sufficient to foster binge-eating behavior and food addictive-like behavior. (A)** Scheme of the experimental procedure of BE model and operant behavior. **(B)** Body weight growth curve of mice exposed to BE model. Mean ± SEM. Repeated measures ANOVA, genotype effect, ∗∗∗*P* < 0.001 CaMKII-CB1-RS vs CB1–KO; ###*P* < 0.001 WT vs CB1–KO. **(C)** Total caloric intake during BE cycles, in the first 2.30 h and 48 h. Mean ± SEM. Student´s t-test, ∗∗*P* < 0.01; ∗∗∗*P* < 0.001. **(D)** Operant responding maintained by chocolate-flavored pellets. Number of reinforcers during 1 h of operant training at FR5 schedule. Mean ± SEM. U Mann–Whitney, #*P* < 0.05 WT/ST vs CB1–KO/ST; ∗*P* < 0.05 CaMKII-CB1-RS/ST vs CB1–KO/ST; §*P* < 0.05 WT/BE vs CB1–KO/ST; α *P* < 0.05 CaMKII-CB1-RS/BE vs CB1–KO/ST; β *P* < 0.05 CaMKII-CB1-RS/BE vs CB1–KO/BE. **(E)** Impulsivity. Number of non-reinforced active responses during the time out period (10 s) in 1 h of FR5 schedule test. Mean ± SEM. U Mann–Whitney, ∗*P* < 0.05. **(F)** Motivation for chocolate-flavored pellets. Breaking point in a 5 h session in PR schedule test. Mean ± SEM. U Mann–Whitney, ∗*P* < 0.05. **(G)** Compulsivity measured by the shock test. The number of shock received in 50 min of the test in which each pellet delivery was associated with a footshock (0.25 mA). Mean ± SEM. U Mann–Whitney, ∗*P* < 0.05.

Based on the CaMKII-CB1-RS mice overeating phenotype, we studied these mice in a binge-eating (BE) model [31,40] (Figure 4A), which mimics human pathological feeding behavior. The model caused overeating when mice had access to a chocolate mixture without affecting body weight, as compared to the corresponding SD-fed mice (Figure 4B–C). Thus, we evaluated the behavior of these mice without body weight differences as a possible confounding factor. During the first 2.5 h of the BE cycle, mice consumed approximately 30% more calories than the SD-fed mice and approximately 70% of these total calories were from palatable food, validating the model (Figure 4C). In this short BE period (2.5 h), CaMKII-CB1-RS mice displayed an intermediate rescue phenotype between WT and CB1–KO mice, while WT mice consumed significantly more calories than CB1–KO mice (Figure 4C).

After 4 BE cycles, we analyzed the operant behavior to develop food addiction. CaMKII-CB1-RS mice previously exposed to BE (CaMKII-CB1-RS/BE) tend to seek more the reward than the rest of the groups in FR5 (Figure 4D), more notorious during the first sessions, confirming their high sensitivity to the reinforcing effects of palatable food. In each food addiction criterion, CaMKII-CB1-RS/BE mice had the highest response as compared to the rest of the groups in the number of non-reinforced active responses during the time out period (10 s) (impulsivity), the breaking point during PR (motivation), and the number of shocks paired with pellet delivery (compulsivity) (Figure 4E–G).

### 3.6 The expression of CB1 in CaMKII + neurons enhances cognitive flexibility and rescues anxiety-like behavior but not depressive-like behavior

Previous evidence showed a negative emotional state associated with the BE model during withdrawal of palatable food [41]. Furthermore, overeating and obesity are associated with impaired cognition (2). Therefore, we assessed cognitive flexibility, anxiety-like and depressive-like behavior in these mice during the withdrawal period.

**Figure 5:**
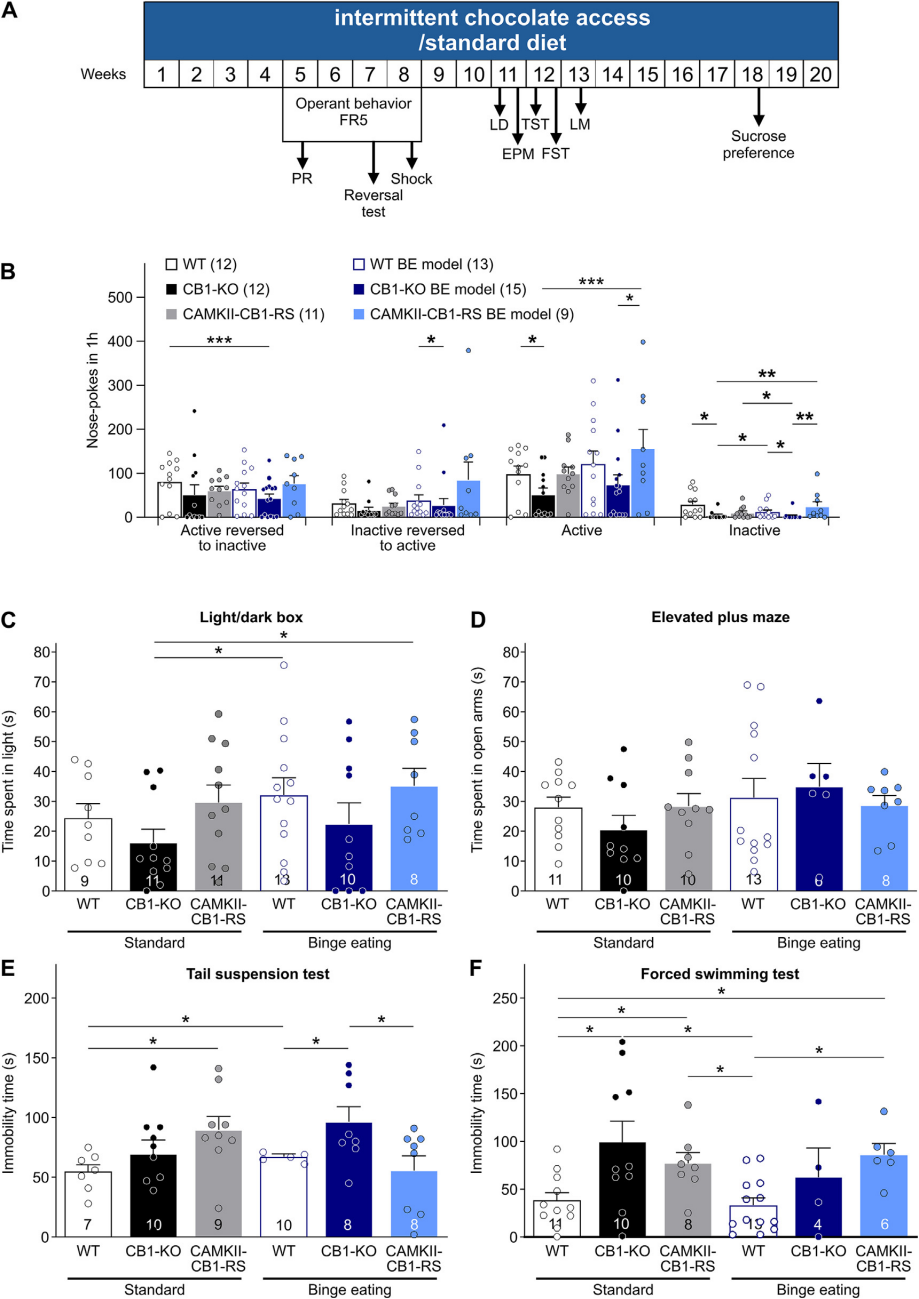
**The expression of CB1 in CaMKII + neurons enhances cognitive flexibility and rescues anxiety-like behavior but not depressive-like behavior. (A)** Scheme of the experimental procedure of BE model and behavioral tests. **(B)** Cognitive flexibility measured by the reversal test and the following session after the reversal test. Mean ± SEM. U Mann–Whitney, ∗*P* < 0.05; ∗∗*P* < 0.01; ∗∗∗*P* < 0.001. **(C)** Light/dark box test. Time spent in the light compartment. Mean ± SEM. U Mann–Whitney, ∗*P* < 0.05. **(D)** Elevated plus maze. Time spent in the open arms. Mean ± SEM. **(E)** Tail suspension test. Immobility time during the tail suspension test. Mean ± SEM. Student’s t-test variances assumed, ∗*P* < 0.05. **(F)** Forced swimming test (FST). Immobility time during the FST. Mean ± SEM. Student’s *t*-test variances assumed, ∗*P* < 0.05.

Cognitive flexibility was evaluated during the withdrawal period in the reversal test (Figure 5A). CaMKII-CB1-RS/BE mice obtained a higher number of active responses in this test than the rest of the groups, suggesting an increase cognitive flexibility, but also a high number of inactive responses (Figure 5B). CaMKII-CB1-RS/BE mice had elevated number of both active and inactive responses during session after the test, confirming an enhanced food seeking behavior (Figure 5B). In addition, 67% of CaMKII-CB1-RS/BE mice did not discriminate between nose pokes (Supplementary Fig. 4).

We also analyzed anxiety-like and depressive-like behavior during the withdrawal period (Figure 5C–F, Supplementary Fig. 5). In the light–dark box, CaMKII-CB1-RS mice, both in control and BE conditions, showed a phenotype similar to WT mice and were less anxiogenic than CB1–KO mice, although no significant differences were observed (Figure 5C). In contrast, no differences were observed before the BE cycle in the elevated plus maze paradigm (Figure 5D). No effect was associated with BE vs control conditions (Figure 5B–C). No significant differences between groups were observed in the number of entries in the light compartment or total number of entries in the light–dark box test (Supplementary Figs. 5A–B), number of entries in the open arms or total number of entries in open and closed arms (Supplementary Figs. 5C–D).

Depressive-like behavior was evaluated using the tail suspension (TST), forced swimming (FST), and sucrose preference test. The BE model had no impact in depressive-like behavior in any genotype (Figure 5E–F), except for WT/BE mice in the sucrose preference test (Supplementary Fig. 5E). Importantly, CaMKII-CB1-RS mice on control conditions showed an increased immobility in the TST and FST (Figure 5E–F) and a reduced sucrose preference (Supplementary Fig. 5E) as compared to WT mice on the same experimental conditions, indicating that CaMKII-CB1-RS mice had a depressive-like behavior in these three tests. None of these behavioral phenotypes were caused by differences in locomotor activity (Supplementary Fig. 5F).

## 4 Discussion

The current unlimited access to energy dense and highly palatable food promotes eating disorders with an unprecedented prevalence [42]. In the light of the obesity epidemic and high incidence of eating disorders urges to investigate the neurobiological mechanisms involved in. Based on our findings, we hypothesize that the expression of CB1 in CamKI + neurons has been evolutionarily selected to shift energy balance towards energy storage by promoting food intake, reducing energy expenditure, and facilitating lipid storage. In particular, we found that CB1 expression on this neuronal subpopulation is sufficient to favor the accumulation of energy by driving overfeeding, food seeking and, under hypercaloric or hyper-glucocorticoid conditions, is sufficient to develop obesity, some features of the metabolic syndrome, binge-eating behaviour and food addiction. In contrast, the selective rescue of CB1 expression in adipocytes has no impact in food intake and only a mild phenotype in obesogenic conditions.

CB1 rescue in CaMKII + neurons drives hyperphagia under caloric restriction conditions, food seeking and palatable food consumption, whereas selective re-expression of CB1 in adipose tissue does not have any effect. In the novelty suppressed feeding test [35], CaMKII-CB1-RS mice showed a reduced latency to approach the palatable food, leading to food seeking and overcoming the natural fear. According to Maslow’s hierarchy of needs, an advantageous evolutionary system that fosters energy accumulation should override other inhibitory behaviors such as safety needs. Starvation or chemogenetic stimulation of hypothalamic AgRP neurons in mice enhanced appetite, but reduced risk assessment, which in turn promotes foraging [43,44]. In the operant behavior, CaMKII-CB1-RS mice confirmed a strong food seeking behavior and obtained the highest number of chocolate pellets in a FR1 schedule, whereas their willingness to work for food reward was not significantly increased. Therefore, selective re-expression of CB1 receptor in CaMKII + neurons strongly promotes overfeeding under caloric restriction likely fostering the replenishment of empty energy storage, and food seeking.

When mice were exposed to obesity models driven by HFD treatment or CORT administration, the rescue of CB1 expression in CaMKII + neurons, but not in adipocytes, is sufficient to largely restore body weight, food intake, and adiposity associated with weight gain, except for glucose intolerance. Notably, CaMKII-CB1-RS mice showed significantly decreased food consumption compared to WT mice despite of a similar body weight, suggesting an additional effect on energy expenditure mediated by peripheral mechanisms. These findings agree with the phenotype of HFD-exposed CaMKII-CB1-KO mice, although no effect on food intake was observed between mutant and WT mice in the previous studies [45]. Thus, the lean phenotype of HFD-fed CaMKII-CB1-KO mice was linked to an enhanced sympathetic nervous system (SNS) tone and a decreased energy dissipation. Energy dissipation through interscapular brown adipose tissue thermogenesis is an important contributor to adaptive energy expenditure in mice and humans [46,47]. Based on this evidence, we suggest that CB1 rescue in pre- or post-ganglionic sympathetic neurons of CaMKII-CB1-RS mice reduces SNS tone and the concomitant decrease in SNS-driven thermogenesis, hence, contributing to the obese phenotype under obesogenic conditions. This hypothesis is supported by the effect of peripheral CB1 blockade (AM6545) in CaMKII-CB1-RS mice. The efficacy of AM6545 treatment in CaMKII-CB1-RS and Ati-CB1-RS mice in reducing body weight and food intake suggests that peripheral CB1 in sympathetic pre- or postganglionic neurons and adipocytes are potential targets in obesity.

Obesity shows many comorbidities including glucose intolerance and insulin resistance. Importantly, CaMKII-CB1-RS mice were partially or fully protected against glucose intolerance in HFD- and CORT-driven obesity, respectively, despite that CB1 rescued mice had a similar body weight as WT mice. This evidence suggests that expression of CB1 in other cell types, most likely in peripheral tissues, are detrimental in CORT/HFD-induced glucose intolerance. Therefore, peripheral CB1 blockade is a promising target to tackle diet- and stress-related type-2 diabetes confirming previous findings [39].

The BE model mimics pathological food intake in humans consuming huge food amount in a short time period [31,40]. When mice had an intermittent access to palatable food, CB1–KO mice consumed less calories than WT mice, whereas CaMKII-CB1-RS mice showed a partial rescued phenotype. Similarly, chronic pharmacological CB1 blockade with rimonabant was effective in reducing BE behavior in female rats [48]. Importantly, BE model did not cause changes in body weight and, hence, we could evaluate the behavior of these mice without body weight changes as a possible confounding factor.

BE studies in rodents demonstrated that an intermittent access to HFD provokes addictive-like responses and changes in the brain reward system that does not occurr when continuous access to the same food [49,50]. The intake of palatable food in a binge-manner mimics the behavior of drug users, consuming excessive drug amount when the drug is available [42]. Under BE conditions, the rescue of CB1 in CaMKII + neurons drives the loss of control over palatable food consumption promoting palatable food seeking, impulsivity, compulsivity, enhance motivation for chocolate-flavored pellets and food addiction. Therefore, our findings demonstrate that CB1 expression in CaMKII + neurons plays a fundamental role in susceptibility to food addiction after prolonged reward exposure. The reward deficiency theory proposes that overconsumption and compulsive-like behaviors are the result of a deficit of the reward system that requires a higher amount of food/drug to achieve similar levels of pleasure resulting in an escalation of consumption [51], which occurs when CaMKII-CB1-RS mice previously have access to palatable food: The reward value is devalued and reward seeking becomes compulsive. Therefore, re-expression of CB1 in CaMKII + neurons seems linked to decreased rewarding value of food triggering overconsumption and compulsive-like behavior.

Our data in CaMKII-CB1-RS mice strongly support that impulsivity may be a predisposition risk factor for obesity, metabolic syndrome, BE behavior and food addiction. Impulsive traits have been associated with compulsive behaviors after prolonged exposure to reward and might increase addictive-like behavior susceptibility [[52], [53], [54]] as well as obesity [55]. Thus, SD-fed CaMKII-CB1-RS mice showed an impulsive behavior in the novelty suppressed feeding test and in the operant behavior. When these mice were under BE, they showed the highest compulsive behavior, corroborating this hypothesis. Interestingly, CB1 rescue in CaMKII + neurons fully restored the weight gain under HDF or CORT exposure. Recently, evidence in humans showed an association between impulsive traits and high body mass index [55]. These data warrant future studies in animal models and clinical studies to investigate the link between impulsivity and pathological eating as well as obesity.

Eating disorders are not manifested as single entities and have a high comorbidity with other disorders including mood and cognitive alterations [56]. This clinical evidence raises the question of a possible overlap between neurobiological circuits that regulate energy homeostasis, reward, cognition and emotions. Accordingly, severe neuropsychiatric side effects have been associated with different types of weight lost therapies [57]. Anti-obesity interventions including rimonabant or bariatric surgery, were associated with a high incidence of psychiatric complications despite its beneficial effect in reducing body weight [20,22,[58], [59], [60]]. Marlow’s hierarchy of needs proposes that behaviors leading to energy storage need to compete with other contingent behaviors, such as safety or mating, for survival based [1]. We propose that overconsumption of food is prioritized over other rival inhibitory needs even in the absence of a caloric deficit. Therefore, we analyzed cognitive flexibility in these mice in a reversal test after BE. CaMKII-CB1-RS mice showed the highest number of operant responding, indicating a strong food seeking behavior and/or impulsivity. We also evaluated anxiety-like behavior in these mice and CB1 re-expression in CaMKII + neurons was sufficient to rescue the anxiogenic phenotype observed in CB1–KO mice [61], although differences were subtle. The anxiolytic phenotype in the rescue mice is congruent with Maslow’s theory and the necessity to inhibit rival inhibitory behaviors, such as anxiety, to promote energy accumulation. In contrast, in the three well-validated paradigms to assess depressive-like behaviors in rodents [62], CaMKII-CB1-RS and CB1–KO mice showed similar responses in behavioral despair and anhedonia indicating that CB1 expression in other cell subpopulations were responsible to cope against these behaviors.

In this study, we focused on elucidating the physiological consequences of a functional rescue of CB1 in CaMKII + neurons or adipocytes on a CB1 null background in terms of food intake, energy homeostasis, and pathological eating behavior. We intentionally chose these two cell types based on the instrumental role of CB1 in these two cell populations in energy homeostasis, as previously described [13,45]. The experimental approaches used in our manuscript clearly demonstrated a rescue of the CB1 function in the two rescue mouse lines. Furthermore, we generated two mouse reporter lines to illustrate the selective expression of the Cre recombinase in adipocytes of Ati-nuGFP mice and mostly in glutamatergic excitatory neurons of CaMKII-nuGFP mice. However, we did not demonstrate that the rescue approach led to CB1 expression at exactly endogenous levels, which represents a limitation of our study. As published in Ruehle et al. [23] and for reasons not fully understood, the presence of the transcriptional stop cassette was not sufficient to entirely block the generation of CB1 mRNA as revealed by quantitative PCR, despite the complete loss of CB1 protein. Therefore, the expression analysis in the rescue mouse lines would need to be at protein and single-cell level, which is beyond the technical approaches presently available. Further studies may be warranted to address these issues.

We only analyzed male mice. The prevalence of eating disorders in humans in consistently higher in females than in males regardless the type of eating-related disorder, hence future studies are warranted in female mice.

The current obesity epidemic and high prevalence of eating disorders urges to investigate the neurobiological underpinnings. This study focussed on the analysis of neurobiological mechanisms underlying overconsumption of food and pathological consequences concurrently with other motivational drives, such as cognitive, anxiety, and depressive alterations. Based on our findings, we proposed that the main function of the CB1 in CaMKII + neurons aims to accumulate energy increasing feeding behaviors, lipid storage and diminishing energy expenditure. In the continuous presence of palatable food or hypercortisolism, this evolved mechanism of survival provides the neuronal underpinnings of obesity, BE and ultimately food addiction. According to Maslow’s hierarchy of needs, if the rescue of CB1 in CaMKII + neurons favours the accumulation of energy, then, other rival motivations needs to be accommodated to achieve its principal role. Thus, CB1 re-expression in these neurons was sufficient to rescue the anxiogenic phenotype of full CB1–KO mice, but not depressive-like behaviors. This study unraveled the different behaviors controlled by the CB1 in this subset of neurons, where the expression of CB1 prioritizes energy storage over other rival inhibitory behaviors. The elucidation of these novel mechanisms provides new insight to unravel the complexity of obesity and eating disorders and new targets to develop safer and more effective treatments.

## CRediT authorship contribution statement

**Elena Martin-Garcia:** Writing – review & editing, Supervision, Methodology, Investigation, Funding acquisition, Formal analysis, Data curation, Conceptualization. **Laura Domingo-Rodriguez:** Methodology, Investigation, Formal analysis, Data curation. **Beat Lutz:** Writing – review & editing, Supervision, Funding acquisition. **Rafael Maldonado:** Writing – review & editing, Supervision, Funding acquisition, Conceptualization. **Inigo Ruiz de Azua:** Writing – original draft, Supervision, Methodology, Investigation, Formal analysis, Data curation, Conceptualization.

## Declaration of competing interest

All authors declare no financial or conflict of interest.

## Figures and Tables

**Figure:**
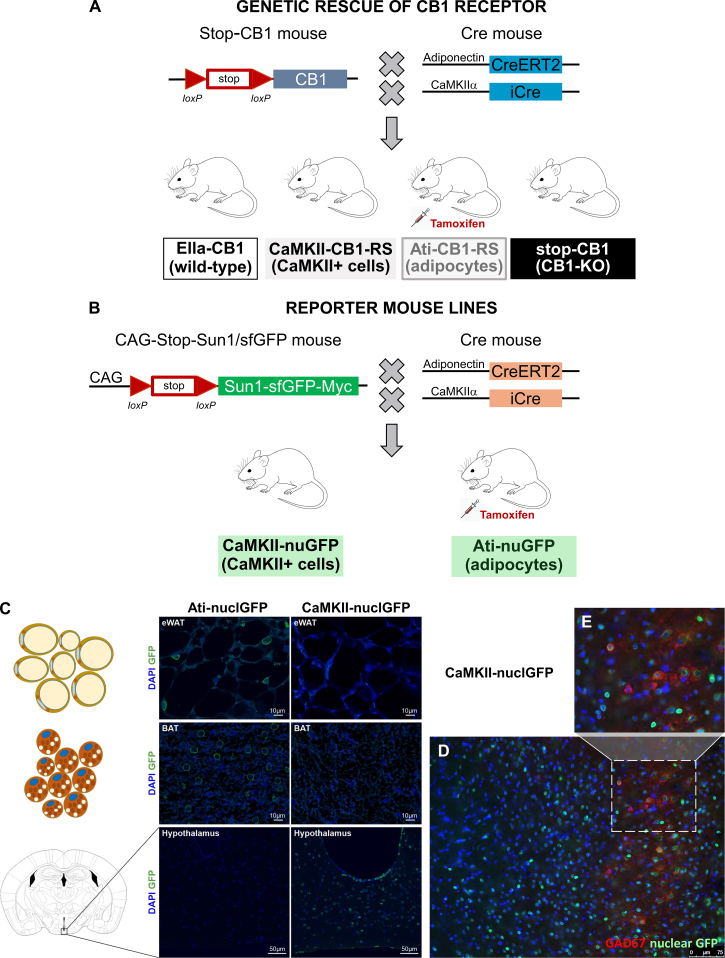
**Supplementary Fig. 1. (A)** Schematic representation of the genetic rescue of CB1 in CaMKII-CB1-RS, Ati-CB1-RS and control (Ella-CB1 and stop-CB1) mice. **(B)** Ati-nuclGFP and CaMKII-nuclGFP reporter mice. **(C)** To validate the genetic approach, reporter mice were generated to analyse nuclear GFP (green) expression in adipose (eWAT and BAT) and brain (hypothalamus) tissue of Ati-nuclGFP and CaMKII-nucleGFP mice. **(D)** Colocalization of nuclear GFP (green) neurons with GABAergic interneurons (labeled as GAD67, red) in the hippocampus of CaMKII-nuclGFP mice. **(E)** Enlarged view of area shown in panel a. Scale bars: 10 (in eWAT and BAT), 50 (Hypothalamus) and 75 μm (in panel a).

**Figure:**
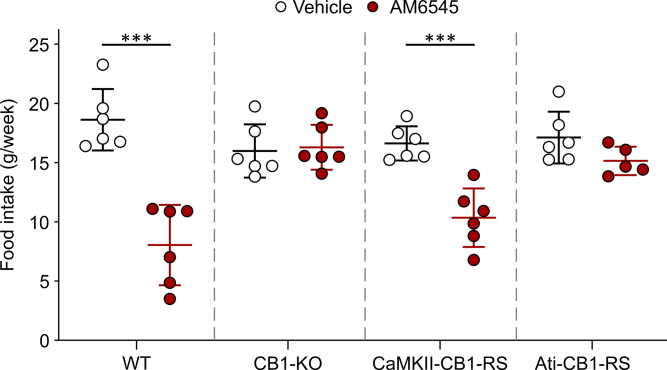
**Supplementary Fig. 2. Food consumption in AM6545 treated HFD-fed mice for two weeks after 8 weeks on DIO model.** Mean ± SEM. One-way ANOVA, multiple comparison, ∗∗∗*P* < 0.001 vehicle vs AM6545.

**Figure:**
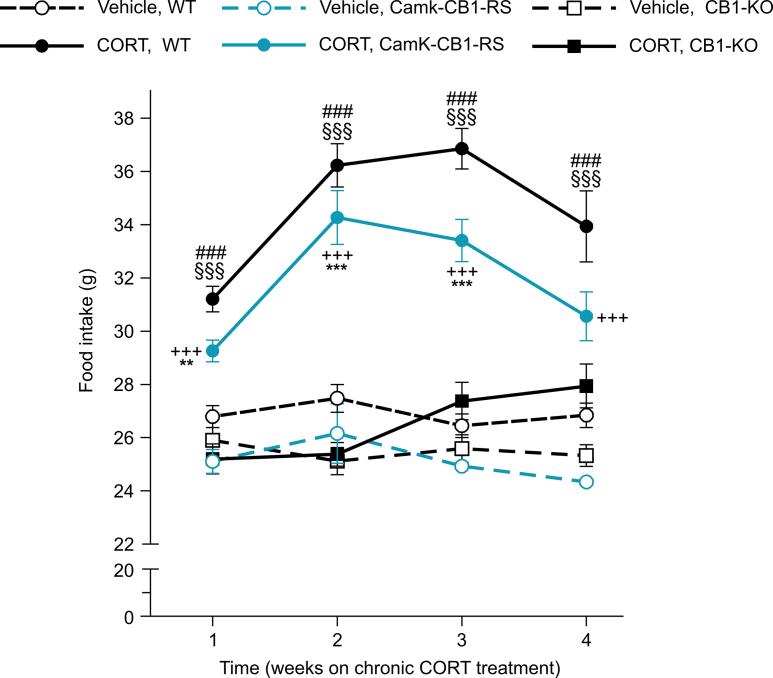
**Supplementary Fig. 3. Weekly food consumption in CORT treated mice.** Mean ± SEM. Two-way ANOVA, and Turkey’s multiple comparisons at indicated time points; ∗∗*P* < 0.01∗∗∗*P* < 0.001, CORT treated CB1–KO vs CORT treated CaMKII-CB1-RS; +++*P* < 0.001, vehicle vs CORT treated CaMKII-CB1-RS; ###*P* < 0.001, CORT treated CB1–KO vs CORT treated WT; §§§*P* < 0.001, vehicle vs CORT treated WT.

**Figure:**
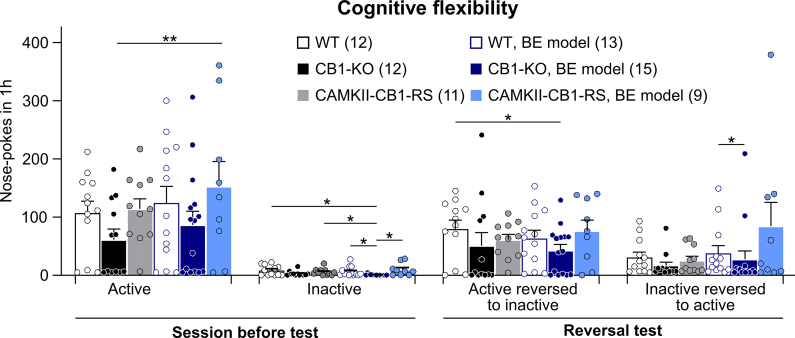
**Supplementary Fig. 4. Cognitive flexibility in CaMKII-CB1-RS mice measured by the reversal test.** Number of active and inactive nose-pokes in the preceding session and the reversal test. Mean ± SEM. U Mann–Whitney, ∗*P* < 0.05; ∗∗*P* < 0.01.

**Figure:**
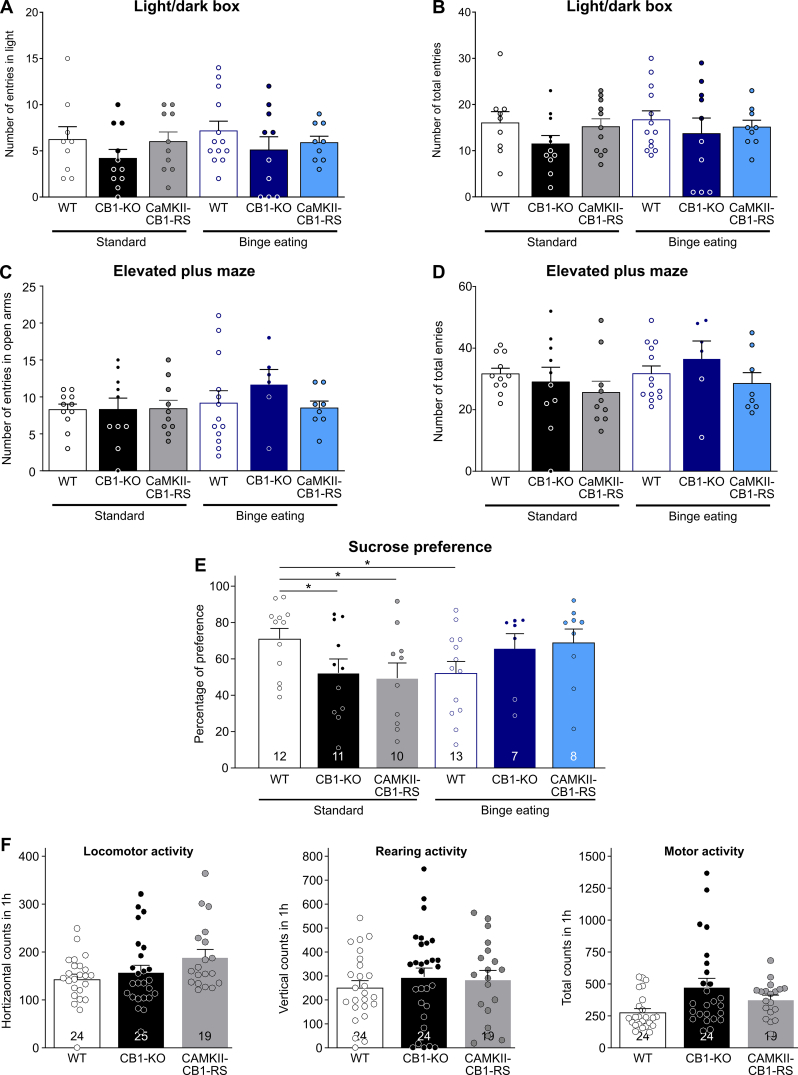
**Supplementary Fig. 5. Anxiety-like behaviors, sucrose preference test and locomotor activity in the BE model. (A)** Number of entries in the light compartment of the light–dark box test. Mean ± SEM. **(B)** Number of total entries in the light and dark compartments in the light–dark box test. Mean ± SEM. **(C)** Number of entries in the open arms in the elevated plus maze paradigm. Mean ± SEM. **(D)** Number of total entries in the open and closed arms in the elevated plus maze paradigm. Mean ± SEM. **(E)** Sucrose preference test. Percentage of preference of 2 days for 2% sucrose. Mean ± SEM. U Mann–Whitney, ∗*P* < 0.05. **(F)** Left panel, locomotor activity measures as by horizontal counts in 1h. Middle panel, rearing activity measures as by vertical counts in 1 h. Right panel, motor activity measured by total counts in 1 h. Mean ± SEM.

## Data Availability

Data will be made available on request.
